# Opthalmia of the New Born*Prepared for the Medical Association of Georgia, April, 1893.

**Published:** 1893-07

**Authors:** S. Latimer Phillips

**Affiliations:** Savannah, Ga.


### OPHTHALMIA OF THE NEW-BORN.*

BY S. LATIMER PHILLIPS, M. D., SAVANNAH, GA.

---

Fully one-third of the cases of blindness collected in the various blind asylums throughout the country is due to this form of eye inflammation, a disease to which the infant is exposed the very moment it opens its eyes after having passed from the womb of its mother. But science has done much in the last two decades in the way of prophylaxis, and one meets not nearly so many of these hopeless cases, sequels to purulent ophthalmia, as formerly.

The disease begins usually about the third day. The lids swell and stick together. At first there is a muco-purulent discharge, finally developing into full purulency. Though generally developing so soon after birth, it has been known to break out on the eighth day. The lids become agglutinated from the discharge drying on the lashes, and when they are separated a thick. creamy matter gushes profusely out. The palpebral conjunctiva is red and thickened, velvety, the ocular congested and oftentimes chemotic. Now, if allowed to go on further in its course, the cornea becomes involved, the disease starting usually as a small gray infiltration, anywhere on the surface of the cornea, gradually spreading and deepening until perforation ensues with all its direful results. When an infant is brought for treatment suffering from ophthalmia, the greatest care should be taken to ascertain the exact condition of the cornea, for upon complete knowledge of the extent of the dis-

* Prepared for the Medical Association of Georgia, April, 1893.

ease depends the treatment and prognosis. I have never known an eye to be lost when I saw the child before the cornea became involved, and I had a faithful nurse. In making such an examination the usual method employed is for the surgeon to hold the head of the infant between his knees, another person sitting opposite, supporting the body of the child. Care must be taken only to make sufficient pressure upon the head of the baby to keep it steady and not to injure it. With Desmarres' lid retractor, separate the lids and scan carefully the whole surface of the cornea. Before this is done all discharges should have been wiped out with pledgets of absorbent cotton wrung out of a solution of bichloride 1 to 5,000. Care should be taken in making the inspection not to press upon the eyeball, or allow the infant to compress the lids too forcibly. I have known cases where there was large destruction of the cornea, the first indication the examiner had of such, was upon sudden compression of the lids upon the eyeball, forcible expulsion of the lens into space. Perforation of the cornea may result simply in adhesion of anterior capsule of the lens to cornea, with more or less cataract, or staphyloma may be so great that the lids cannot be closed over it. Usually if the perforation has been central, and atropia used in time to draw back the iris, the result will be simple adhesion of the parts in contact; if peripheral more or less staphyloma. One eye may be effected, the other escaping, but generally both become innoculated at the same time with the disease. While the disease apparently is of the same virulence in the two eyes, often one cornea is perforated and the other remains intact.

In earlier times, "taking cold" played a very important role in the disease of ophthalmia of the new-born. Prof. Benedict,* of Breslau, in 1814, speaks of taking infants, a few days after birth, in the winter time, to the church to be sprinkled with cold water, as a cause, and even so late as 1870, Prof. Junken† speaks of the exposure of the infants head, free of any cover as a frequent and important cause. But in England as early as 1807, Gibson, and in 1832, McKenzie traced the cause to its

* Handbuche der Augentzundungen.
† Augendiatetik, p. 43.

true source, the vagina of the mother. In the great majority of cases, upon investigation, the mother will be found to have "whites," from some cause or other. A bright light was thrown upon the subject when, in 1879, Neisser discovered the same kind of cocci in the discharges from the eyes of the infant, as in those from the vagina of the mother and urethra of the father. The gonococci can gain admission to the eyes of the child during parturition, and after its birth. In the former the microbes enter the conjunctival sac during the passage of the head through the vagina, the lids becoming separated. Especially is this to be expected when the labor has been a tedious one. When contagion results after the birth of the body, it is usually from carelessness or ignorance on the part of the one providing for the wants of the child. When bathing the body of the child some of the discharge from other parts of the body may be splashed into the eyes of the little one, or the sponge or cloths employed in cleansing other parts of the body used about the eye, or even the hands of the nurse or physician may convey the poison. Very naturally this disease is found most frequently among the poor and in dispensary and hospital practice, but occasionally it is found in the best private practice, for we do not by any means find here perfection.

*Prophylaxis.*—That we may appreciate better the frequency of the disease and the importance of prophylaxis, I will introduce the following table of statistics of its occurrence in lying in hospitals, collected by Prof. Haussmann :

| INSTITUTIONS. | Years through which the Statistics were Gathered. | Percentage of Blenorrhoea. |
|---|---|---|
| Berlin University, lying in Institute | 1829—1869 | 1.1— 8.3 |
| Berlin Charity | 1817—1879 | 7.4—21.3 |
| Breslau | 1827—1877 | 7.0—18.5 |
| Dresden | 1826—1875 | 2.2—25.3 |
| Halle | 1840—1879 | 2.8—21.7 |
| Leipsic | 1849—1879 | 7.6—13.6 |
| Munich | 1860—1881 | 0.8— 5.2 |
| Stuttgart | 1828—1879 | 5.8—20.9 |
| Vienna Clinic 1 | 1857—1864 | 0.8— 2.3 |
| Vienna Clinic 2 | 1857—1864 | 0.6— 1.6 |
| St. Petersburg | 1845—1854 | 1.2— 1.4 |
| Stockholm | ......... | 2.9— 7.9 |

Before the child can have a blenorrhœa the mother must have it, and with her it is usually gotten from the father. So prophylaxis really begins with the father. A man suffering from gonorrhœa in any form should not have intercourse with his wife, but as we cannot guard against such contingencies, we have to confine our care to the proper disinfection of the vagina before the birth of the child, and the care of the infants eyes afterward. It is said a single gonococcus from the urethra of the man is sufficient to set up a long continued gonorrhœa in the woman. Hermann Cohn* mentions an interesting case. A young man married an intact virgin. She developed, during thc honeymoon, a light fluor albus. The man had been suffering from a long standing gonorrhoea, with very slight symptoms. The first baby developed on the third day a blenorrhœa of the eye. Dr. Cohn said to his assistant the next year after that, when he read in the papers of the expected confinement of this lady, lookout for another case of blenorrhœa, and so it was with second, third and fourth child.

Before the birth of the fifth, the vagina was washed out with a solution of carbolic acid, and the child escaped blenorrhœa. When men with chronic gonorrhœa marry, very special care should be taken by the physician to guard against any eye inflammation in the infant during and after delivery. In every case where, during pregnancy, there has been a purulent discharge, or even a severe mucus one, from the mucous membrane of the vagina, copious washing or syringing of the latter cavity should be undertaken with a weak solution of bichloride or carbolic acid before the labor begins.

Even after the baby is born it may contract the disease, some of the cocci having gotton entangled in the lashes, or on the lids, and when cleansing the eye forced in. So the greatest care should be taken in this respect, to use fresh cloths, or better pledgets of absorbent cotton wrung out of a solution of bichloride, in cleansing the parts about the eyes. Numberless antiseptics have been employed by numberless physicians to bathe the eyes with, and this care has been the means of pre-

* Lehebuch der Hygiene des Auges, p. 50.

venting much inflammation of the eyes, thus reducing, in a great measure, the percentage of this disease. Perhaps the method of prophylaxis most extensively employed, is that of Crede', which consists of dropping into the eyes of each infant as it is born, one drop of a 2 per cent. solution of nitrate of silver. In large lying in institutions where the weak and uncleanly are brought in large numbers for care during the lying in stage, this method is invaluable. Crede' had only one case of ophthalmia neonatorum in the course of three years, in 1,160 children where this method was used. Other writers have had similar experience. Haah* collecting statistics from various institutions found that before the introduction of Crede's method, out of 42,871 infants, 8.9 per cent. had blenorrhœa, afterward the percentage was reduced to 1 per cent. In the great European clinics, since its introduction cases of phthisis bulbi, staphyloma, leucoma, etc., have very much diminished in numbers. As a means of protection against this disease, in some cities cards have been printed and distributed to all known practicing midwifes, and placed in the dispensaries, calling attention to the danger a child's eyes are exposed to when this disease developes, and urging that immediately upon such symptoms, such as mattering of eyes and swelling of lids develope, the child be taken to a physician. The frequency of the disease is pointed out, and its direful results if not properly treated. By this means many an eye, which otherwise would have been lost, has been preserved, and a useful human being retained who might otherwise have become a burden upon a community.

* Hermann Cohn, Lehrbach der Hygiene des Auges, p. 54.

of the solution, every time the cleansing process is gone through, and then destroyed, fresh ones being employed every time. Care should be taken that the soiled pledgets are not flung about the apartment and thus become the means of inoculating some other eye. Just before using the cotton it would be well to inject into the conjunctival sac some of the solution of same strength as above, with ordinary eye pipette. Instead of the bichloride we can use a saturated solution of boracic acid, or a weak solution of potassium permanganate, just sufficient to color the water a pale claret. As there is much swelling of the lids and congestion, cold applications become very useful. The usual method of applying cold is by keeping strips of linen on ice and renewing every few minutes, or wringing them out of ice water and then applying. They very soon absorb the heat from the lids, and if not renewed immediately become hot and act as a poultice, thus doing more harm than good. In very severe cases I prefer to use crushed ice in layers of muslin, laid on the lids. This reduces the temperature and keeps it down, and thus lessens the suppuration.

Chemosis is a marked symptom, and it is this which threatens the cornea; if we can control it, we usually have the disease in check, and cold is a great factor in diminishing it. I have never found it necessary to scarify the hypertrophied and intensely congested conjunctiva, for, in many cases, any unusually rough handling will produce hemorrhage, and I do not know that it is beneficial. Nor do I deem it necessary to split the lids at the outer canthus as advised by the text-books, the diminution of the blood supply to the cornea is due not so much to the pressure of the lids, but to the chemosis about the eyeball. The case must be rare that calls for this procedure. I look upon nitrate of silver in moderate strength as the great sheet anchor in this disease. From the very beginning of purulency to the time it disappears, I use it, and have yet to see harm. Even when ulceration of the cornea has taken place I employ it, and feel perfectly safe in doing so. I have never yet found it necessary to use, in this disease, a stronger solution than gr. v. to the ℥i. though in gonorrhœal ophthalmia of adults I have used it as strong as to x gr. to ℥i. If we can get the desired effect from a weaker solution, what is the use of employing a stronger? I am inclined to think those who have had trouble with nitrate of silver, have been those who used the strong solution. Besides using a weak solution, I do not deem it, in the majority of cases, needful, to cauterize the conjunctiva more than once in twenty-four hours. There will be exceptional cases where the disease is very severe when it will be necessary to use it twice. As to the method of applying it, that which has proved the most satisfactory in my hands is the following: Two or three drops are dropped into the conjunctival sac with an ordinary drop tube, the lids being held open by the thumb and forefinger of the surgeon. The child generally in making efforts to close the lid when thus held, will cause them to become everted and the palprebral conjunctiva thus exposed. The solution thus reaches the parts desired to be cauterized. If the conjunctiva is not thus exposed, the solution dropped between the lids, by the free movements of the eye reaches all parts of the conjunctival sac, and if any gets upon the cornea it is harmless, it being not sufficiently caustic to destroy this tissue, as it is much less easily affected than the inflamed and sensitive papillæ of the conjunctiva. It is not always an easy matter to evert the lids and apply the silver with a brush, or the mitigated stick, as advised by some. Often the lids being too much swelled and brawny to admit of this. But by using it as advised above we can always get it in. After applying the silver we will notice a whitish appearance of the surface of the conjunctiva wherever it has come in contact. This is soon thrown off and leaves the surface below in a more healthy condition. The amount will vary according to the strength of the solution. It is well to have at hand a solution of common table salt, to inject between the lids if the burning appears to be too severe. A drop or so of cocaine will ease the pain that follows for a while. Immediately after the cauterization cold applications should be renewed. If ulceration of the cornea has taken place, atropia, gr. ii. to ℥i. should be used if it is central; eserine, gr. ½ to ℥i.

if peripheral. These remedies are used principally for their mydriatic and myotic effects. The former pulling the iris away from the point of ulceration, thus preventing any adhesion of the iris and cornea, besides it has a very soothing effect in ulceration of the cornea. The latter contracting the pupil draws the iris away from periphery and guards against a hernia.

Even if ulceration of the cornea should have taken place before the physician saw the case, much may be done to preserve the sight to a more or less extent. I should not advise paracentesis or opening the anterior chamber in threatened perforation, for we only admit the germs into the interior of the eye, and gain nothing by it.

